# Integration of film and game: multi-case study on narrative strategies in Hollywood movie gamification

**DOI:** 10.3389/fsoc.2025.1534556

**Published:** 2025-03-25

**Authors:** Zhuxin Fang

**Affiliations:** Faculty of Arts, Humanities and Cultures of University of Leeds, Leeds, United Kingdom

**Keywords:** gamified movie, narrative method, narrative mechanism, immersive film, aesthetic transmutation

## Abstract

In order to explore the innovative approach to the integration of films and games, and to provide theoretical support and practical guidance for the development of gamified films, this essay uses the method of multi-case study to systematically sort out the unique gamified narrative strategies of three Hollywood films, *Ready Player One, Free Guy, and Everything Everywhere All at Once*, as well as the transmutations of aesthetics brought about by these narrative strategies, in terms of the dimensions of narrative approach, narrative mechanism, and immersive design, and so on. It is clear through the study that the narrative strategies of gamified films have had a profound impact on film creation and the film industry. It not only promotes the continuous innovation of film narrative structure and presentation methods, but also promotes the expansion and integration of the film and game industries.

## 1 Introduction

With the rapid development of computer graphics technology and virtual reality technology, media integration has become an unstoppable trend of the times. Gamification is becoming part of our life, and with the growing technology and digitalization, it has become a prevalent part of different user activities (Tayal et al., 2022). Cinema and games, two influential forms of popular culture, have gradually broken down the boundaries between each other in this context, presenting a trend of in-depth integration. In recent years, as an innovative product of the integration of film and game, gamified film has emerged in the field of film and television and received widespread attention. The uprising of gamified film has not only injected new vitality into the film industry, but also challenged the narrative strategy of traditional film.

Gamified movies refer to movies with game features. Galloway (2006:62) first proposed the concept of gamified movies in his book to refer to phenomena that incorporate specific game forms into film grammar. Kallay (2013:1) used a similar term ‘game movies’, believing that digital culture was reshaping the narrative, structure and visual aspects of cinema. Gamification is a process of using or applying game-related elements to solve problems in the storyline. It involves using game design elements, game thinking and game motivation to attract audiences and solve problems in non-game environments. The purpose of movie makers using gamification strategies is to cultivate players’ or users’ interests, increase their participation, or guide certain behaviors (Risso and Paesano, 2021). Obviously, the gamification of movies is the intersection of games and movies, and the conceptualization of gamification is guided by the influence of computer games on the narrative of movies (Larsen, 2019). A movie incorporates a large number of game narrative methods, such as non-linear storytelling, protagonist involvement in multiple tasks, and multiple world lines. Meaning takes a vital role in a game narrative, which is well-designed as the greatest appeal to the target audience (Naul and Liu, 2020). Gamified film narratives can create a greater sense of interaction with the audience, going through game missions or traveling through the multiverse with the film’s protagonists(Gallon, 2021).

Gamified movies integrate game elements and mechanisms into the narrative process, construct and convey the story based on game design ideas and principles, and focus on three major gamified narrative strategies. Firstly, they use a multiverse narrative. Utilizing game props and completing the final game task has three major features: non-linear mechanism, save and revive mechanism, and time constraint mechanism. Secondly, they construct an avatar mechanism. The protagonists in game movies usually have many suppressed desires in the real world, which can be realized through avatars in the multiverse. Thirdly, they create visual wonders, including gamified visual elements, gamified cinematic language, gamified visual effects, and gamified textual intertextuality (Shu, 2023).

How does the gamification strategy empower the narrative or dissemination of movies? Arsenopoulou et al. (2023) studies’ have analyzed the role of narrative in promoting game-adapted films, exploring the impact of electronic games on the film narrative, following the “electronic game logic” described by Bakran and the film narrative mode of electronic games. The common features and overlaps between these two narrative forms have been studied from the early stages of electronic game production; exploring the cinematic characteristics that can be traced back to earlier and newer electronic games, with a focus on issues such as scene, editing, genre, and materiality. It is believed that narrative forms play an important role in promoting the artistic effect of gamification (Arsenopoulou et al., 2023).

In summary, in recent years, the research on gamified film narrative has achieved certain results, not only has a more explicit expression of the concepts of gamification and gamified film, which defines the scope of the research on gamified film narrative strategy, but also has formed a clearer classification on the research of narrative strategy types, and has deeply analyzed the impact of gamified film narrative strategy on the film’s narrative and dissemination ability. However, the research still has some shortcomings. Firstly, the current research on gamified film narrative strategies is relatively one-sided, failing to systematically sort out and summarize them. Secondly, current research often focuses on form rather than content, and the details of gamified narrative strategies are not clear. Furthermore, the aesthetic transmutation brought about by gamified film narrative strategy has not been thoroughly analyzed and discussed. Based on this, this essay uses the method of multi-case study to select three Hollywood gamified films *Ready Player One*(2018), *Free Guy*(2021), and *Everything Everywhere All at Once*(2022), to discuss the narrative strategy of gamified films from the aspects of narrative technique, narrative mechanism, immersive design and aesthetic transmutation. It is expected that this study can promote the deep integration of film and game industry, and enrich and improve the film narrative theory system.

## 2 Abundant narrative approaches

Gamification film often breaks the traditional linear narrative of the film, using non-linear narrative, multi-clue narrative, circular narrative, meta-universe narrative and other techniques to make the film more complex, interactive and interesting, to provide the audience with more space for exploration and imagination, and simultaneously to enhance the film’s artistic innovation and experimentation.

### 2.1 Non-linear narrative

The non-linear narrative of film breaks the traditional narrative mode of “smooth narrative” in the past, and is now an important exploration and innovation in the history of cinema (Lin et al., 2023). Non-linear narrative no longer follows a single, linear storyline, but instead creates a story that is full of variables and surprises, and can show fragments of the story at different points in time, so that the audience can gradually understand the whole story in the process of piecing together these fragments. This kind of narrative can enhance the complexity and depth of the story, bringing the audience a more innovative and informative viewing experience.

*Ready Player One* cleverly employs non-linear narrative techniques, breaking the traditional narrative framework, through the tightly intertwined reality and the virtual world of ‘Oasis’, bringing the audience into a virtual world full of infinite possibilities. The timeline of the film is not a single linear development, but full of jumps and flashbacks, which makes the narrative more flexible and changeable. For instance, the main character, Wade, constantly reminisces about Halliday’s past and his love for the game as he searches for the egg. These memories not only reveal Halliday’s inner world to the audience but also add a nostalgic and sentimental touch to the film.

*Free Guy* uses a non-linear narrative to build a double narrative space–time between in-game and out-of-game. The in-game storyline revolves around protagonist Guy’s adventures and growth in the game world Free City, while the out-of-game storyline focuses on real-world game designer Millie’s investigations in order to find out the evidence of the stolen game code. These two story lines intertwine with each other through detailed ambiguities and parallel montage techniques to form a complete narrative closed loop, realising free switching between in-game and out-of-game, and enhancing the tension and attraction of the narrative.

*Everything Everywhere All at Once*’ constructs the film plot through non-chronological sequences such as time retracing, time repetition and time jumps, aiming to break the audience’s expectations and show the chaos and unpredictability of the real world, bringing a broader perspective, richer content and more objective expression to the text of the film. The audience’s sense of engagement and desire to explore is heightened as they piece together and understand the full picture of the story. For example, whenever Evelyn’s conflict with her daughter escalates, the scene suddenly cuts to footage of her youthful elopement, suggesting that she subconsciously longs to retrace major life decisions. Another example is the repetitive editing of actions such as folding towels and issuing invoices in the laundromat, which, together with the quick jump cuts, reinforces the sense of meaningless repetition of middle-aged life.

### 2.2 Multicue narrative

Multi-clue narrative refers to the fact that when a film tells a story, it unfolds it by using multiple parallel clues, which may involve different characters, places or events, which are intertwined with each other and together form the narrative framework of the film. This narrative method can enrich the plot and content of the film, making the story more three-dimensional and diversified.

*Ready Player One* adopts a multi-clue cross-parallel narrative mode, which makes the film more intriguing. The main character Wade’s egg hunt journey and other players’ adventures are interspersed with each other, which enriches the story content and enhances the sense of narrative rhythm. Meanwhile, the alternating use of linear and non-linear narratives adds layers and depth to the film, such as the interweaving of Wade’s memories and Halliday’s past, presenting the audience with a future world full of imagination and creativity.

*Free Guy* has several intertwined story lines. In addition to the two main lines, in-game and out-of-game, there are also spinoff stories such as the programmer entering the game to hunt down Guy, and the player broadcasting live in the game. These story lines are intertwined and together they drive the plot.

### 2.3 Circular narrative

A circular narrative is a type of narrative that emphasises themes or emotions by repeating an event or episode in a circular pattern.

In *Free Guy*, the main character, Guy, starts out as an NPC in the game without his own consciousness, living the same boring life repeatedly every day. When he awakens and tries to change himself, everyone is puzzled and questions him, so much so that he has to act according to a prescribed procedure. This cycle allows the viewer to feel his helplessness and pain, as well as his struggle to get out of the current stage of his life, triggering the viewer to think about his own destiny.

### 2.4 Metaverse narrative

According to the Wall Street Journal, the metaverse is “a virtual world where our digital avatars and those of people in our communities and around the globe come together to work, shop, attend classes, pursue hobbies, enjoy social gatherings and more” (Needleman, 2021).

Yu (2021) points out that ‘virtual worlds with synchronicity and a high degree of mimicry are fundamental conditions for the composition of the metaverse’.

The application of metaverse technology in the film industry has triggered profound changes in both film narratives and audience interaction. This transformation not only enriches the narrative structure of films but also deepens the immersive experience for audiences, creating a new dimension for film as a narrative medium (Dai, 2024).

Gamified films often draw on the concept of meta-universe to present one or more virtual spaces parallel to and interacting with the real world through narrative. This space is full of imagination and infinite possibilities, and the audience can experience the adventures, challenges, and interactions in it through the characters’ perspectives, and at the same time trigger thoughts on the boundaries between the virtual and the real, the future of science and technology, human civilization and other issues.

The world of Oasis in *Ready Player One* can be seen as a vivid display of the meta-universe. In 2045, the year in which Wade lives, the real world has fallen into the predicament of material scarcity. In the ‘Oasis’, a virtual world parallel to the real world and full of infinite possibilities and imagination, countless people can not only escape from the reality, but also socialize and take risks through their virtual avatars, so as to realize their own dreams and aspirations.

Through vivid storylines and visual effects, *Free Guy* shows viewers a possible form of the meta-universe, i.e., a world where reality and the virtual are intertwined and full of infinite possibilities. Using technical manipulation and specific viewing angles to build the virtual world toward its real form, the film uses unique narrative perspectives and plot threads to demonstrate the unique threads and intersections between the virtual and the real, as well as the interactions between the two (Zhu, 2022).

*Everything Everywhere All at Once* uses a gamified narrative approach to innovatively present the space of the multiverse. Although the protagonist of the film is not in the game space, the narrative structure of the multiverse reflects the narrative logic of the game design, and the game mission, game rules, and NPCs issuing the mission are all implied in the film. The mission of the game is for the heroine Evelyn to save multiple parallel universes, and the rules are that Evelyn needs to perform specific actions in order to connect with herself in different parallel universes, thereby acquiring special abilities and strengthening herself so as to complete the mission. By traveling between multiple universes, Evelyn resolves the crisis on Earth, and at the same time re-examines and re-experiences her life under different choices in the process. This multiverse setting not only provides the film with rich narrative layers, but also allows the audience to follow the protagonist to explore the possibilities of different life paths.

## 3 Multiple narrative mechanisms

Gamified films often use games as the underlying logic, integrating some of the core concepts of games, building frameworks, telling stories, and creating worlds through carefully designed narrative mechanisms, emphasising the audience’s sense of participation and interactivity, and making the films with a fantasy or sci-fi overtone, more attractive and fluent.

The narrative mechanism of the gamified film is a comprehensive concept, which includes multiple aspects such as theme, content, character perspective, level missions and reward mechanism, etc. These elements are intertwined and work together to constitute the unique narrative style and charm of gamified film.

### 3.1 Gamification settings for themes and content

The themes and contents of gamified films play a core function of narrative in setting the tone of the story, conveying the core ideas, and triggering emotional resonance in the audience. Deeply influenced by digital games, the themes and contents of gamified films are usually closely related to digital games, which can be broadly classified into three categories as of now. First, the characters travel and interact between the game and reality, exploring the intersection of the two. Secondly, characters face challenges and growth, and through game elements such as adventure, combat, and escape, the audience is presented with an exciting storytelling experience. The third is the combination of profound themes with the entertainment of the game, making the film both thought-provoking and entertaining.

#### 3.1.1 The intersection of game and reality

The intermingling of game and reality is one of the important themes of gamified films. The combination of game elements and the real world not only enriches the form of film expression and brings new development opportunities for the film industry, but also brings the audience an unprecedented viewing experience and richer and more diversified entertainment choices.

*Ready Player One* utilises the elements of games to not only connect them closely to reality, but also to explore the boundaries between them. The VR device connects these two worlds, the transition between which is accomplished by the main characters putting on and taking off the device. The main characters are unpopular in real life but highly capable in the game world. The main character team is tested by real-world problems as they continue to pass levels in the game. The biggest villain in the film is constantly sniping at them in reality, which means that they are faced with the dual challenge of juggling real-life crises as well as getting through the game. For instance, when the main character’s team is being pursued by Nolan’s team in real life, the main character is affected by real-life factors such as being crashed into, which causes the corresponding in-game character to be incapable of completing the required actions and thus accomplish the in-game mission, which reflects how the progress of the main character’s in-game mission is affected by reality. The film ends with the main characters succeeding and gaining control in the game, ultimately changing the real world through their victory therein. This deep intermingling of game and reality triggers the audience to think deeply about the relationship between the real world and the virtual world, a common and important theme that is typical of gamified films.

#### 3.1.2 Character challenges and growth

Challenge and growth is a central and pervasive theme in gamified films. Films often show the growth of the main character by setting up a series of challenges, so that the audience can more intuitively understand the close connection between challenge and growth, and be inspired and motivated in real life. These challenges might be adventures, battles or puzzles to be solved in the virtual world, or they might be obstacles and difficulties to be overcome in the real world. In the course of the challenges, the main characters experience failure, frustration and pain, but it is these experiences that teach them how to face difficulties, how to persevere in the pursuit of their goals, and how to find hope and opportunity in the face of adversity.

*Free Guy* is centred around the theme of challenge and growth and shows Guy’s self-awakening and transformation in the gaming world. As Guy gradually develops self-awareness, he begins to question the rules of the game world and eventually chooses to accept and integrate into this virtual world. In this process, Guy faces challenges from both the game world and the real world. He needs to deal with hostile players in the game world, deal with the conflicts that exist in virtual reality, and at the same time face his own internal struggles. Through constant challenges and efforts, Guy gradually grows into an individual with autonomous consciousness and emotions. He learns how to interact with the players and how to change the rules of the game world. This process of growth not only reflects Guy’s personal courage and determination, but also signifies that everyone has the potential for self-transcendence in the face of difficulties and challenges.

#### 3.1.3 Profound themes combined with the entertainment of the game

In the traditional impression, gamified films often give people the impression that they are over-entertaining and under-artistic. This impression may stem from the fact that in the early development stage of gamified films, some works overly pursue sensory stimulation and entertainment effects, while neglecting the artistry of the film. With the development of film art and the advancement of technology, gamified films are constantly exploring and innovating. Some works begin to try to combine game elements with film art, which not only retains the interactivity and fun of the game, but also enhances the artistry and narrative depth of the film.

*Everything Everywhere All at Once* explores topics related to family relationships, self-worth and life choices through the story of its main character, Evelyn, demonstrating the film’s exceptional narrative skills and the director’s humanistic approach. The embodiment of NPCs is Evelyn’s husband Waymond, whose first change of temperament allows Evelyn to realize that she is a character in one of the countless parallel universes. He teaches her how to connect with her parallel-universe-self, acquire different abilities, gradually learn to save the world, and eventually try to reconcile with her daughter. These plots not only reflect the logic of the gamified narrative, but also provoke the audience to think deeply about the nature of life and the meaning of existence. The cinema has attracted widespread cultural attention globally, and its unique cultural perspective and exploration of themes such as family and emotions have triggered resonance among viewers from different cultural backgrounds. Its remarkable success demonstrates that gamified narrative techniques and the concept of multiverse can be combined with profound themes to convey cultural values and emotional connotations of universal significance through innovative narrative and presentation methods, providing an example for the pursuit and breakthrough of gamified films at the artistic level.

### 3.2 Gamification of the main character’s perspective setting

Characters in gamified films are often given gamified settings with complex, interactive correlations between the characters. They can have specific skills, attributes, or tasks like the characters in the game, and this setting makes the film more interactive and interesting, which can attract the audience’s attention and trigger their empathy. The setting of the main character is undoubtedly the most crucial, which not only guides the audience’s cognition, promotes the development of the plot, and creates emotional resonance but also makes the audience feel as if they are in the virtual world constructed by the film, which is one of the important narrative mechanisms of gamified films. As for the current situation, the main characters of gamified films are usually gamers, awakened NPCs (non-player character), explorers, or puzzle solvers. Certainly, these genres are not mutually exclusive, and a main character may feature multiple genres at the same time. Furthermore, as gamified films continue to evolve and innovate, the types of main character identities may become even richer and more diverse.

#### 3.2.1 As a player or gamer

The main characters of gamified films often appear directly as gamers, reflecting the deep fusion of game and reality that is the typical scenario of gamified films. They can be anyone in the real world who somehow enters the virtual game world and embarks on a series of adventures and challenges.

*Ready Player One*’s main character is game player Wade, a man who is unattractive in real life but capable and charismatic in the gaming world. He goes through many difficulties with his friends set by the founder of the test, getting him left in the oasis of the egg, ultimately preventing Sorrento, head of the tech giant IOI, from profiting from the oasis and inheriting the legacy of the founder of the story. The film cuts into the storytelling from his point of view, connecting the game world to the real world, and is able to show the audience the growth and eventual success of the main character in his actions to save both worlds.

#### 3.2.2 As the game’s awakened NPC

Gamified cinema also cuts into the storytelling from the perspective of an NPC, connecting the game world with the real world, breaking through the player-driven model, and enabling the audience to think about the rules of the game world, its significance, and its connection with reality from different dimensions, which is of great significance to the study of narrative innovation in gamified cinema.

*Free Guy* aims to tell the story from the perspective of NPCs, breaking the classic sense of the hero’s main character, letting the traditional supporting role become the master of discourse, and re-examining the game world through the non-traditional perspective of NPCs, creating a novel visual presentation and entertaining narrative effect (Fan and Wang, 2021). The main character, is an NPC who follows a set routine every day. One day, he awakens to the realization that he is just a background character in the game Free City, but Antoine, the owner of Freedom City, is planning to shut down the game in order to launch a new one. Everyone is set to disappear, and only Guy has the power to save them all. Guy and Millie, a real-life gamer whose game data was stolen by Antoine, team up to save the game and let all NPCs live by their own rules.

#### 3.2.3 As an explorer or a puzzle solver

The main character of a gamified film can also be an explorer or a puzzle solver. They have exploration or puzzle solving as their main task, usually with extraordinary abilities or skills, and need to search for clues in a complex space–time setting, break through a series of levels, solve mysteries, defeat enemies, and advance the story.

Evelyn, the main character of *Everything Everywhere All at Once*, possesses the extraordinary ability to travel through the multiverse and is a character who is both an explorer and a puzzle solver. Her experiences in different universes are comparable to exploring and solving puzzles in a game. Under the complex space–time setting, unknown clues are hidden in all corners of the multiverse, and Evelyn is like exploring in a huge game map. Entering the story from such a perspective brings a unique way of constructing the film, and provides an excellent example for studying the innovation of non-traditional gamified film narratives.

### 3.3 Design of level tasks and reward mechanisms

Based on the logic of video games, gamified films often transform traditional linear narratives into more interactive and structured forms of expression through the design of level missions and reward mechanisms, becoming one of their key narrative methods. The story is split into segmented plot units similar to game levels, with each ‘level’ corresponding to a specific scene, mission objective and conflict escalation, and virtual rewards stimulate the audience’s sense of participation, transforming conventional passive viewing into active exploration and enhancing the narrative appeal.

#### 3.3.1 Level tasks and rewarding mechanics in *Ready Player One*

The level quests and reward mechanics in *Ready Player One* are an important part of its narrative and gamification experience, and these designs not only drive the plot, but also deepen the film’s themes and strengthen the narrative tension. Each level in the film hides a variety of clues, puzzles, and unexpected challenges that not only test the player’s skill, intelligence, and teamwork, but are also filled with homages to pop culture. Players continue to overcome level after level, never giving up even in the face of difficulties, and the rewards they receive for their efforts not only excite and satisfy the viewer, but also reflect an exploration of endeavor, co-operation and the balance between the virtual and the real.

Details of the level missions and reward mechanisms in *Ready Player One* are shown in the table below (Table 1).

**Table 1:** List of level tasks and reward mechanics in *Ready Player One.*

Level tasks/ reward mechanism	Content
Level tasks	Level 1: Players are asked to face a variety of obstacles and competitors, including broken roads, collapsed bridges, malicious competitors and vicious gorillas. The main character, Wade Watts, manages to find the secret to clearing the level through a unique strategy - stepping back and looking within himself. This level not only tests the player’s courage and intelligence, but also signifies that sometimes in life you need to take a step back in order to see the whole picture.
	Level 2: Relating to Halliday’s regrets, the player needs to retrace his steps, escape the past, and complete the step not taken. Wadsworth discovers clues in this level about Halliday’s first girlfriend that he missed out on due to his cowardice and timidity, and manages to get the second key. This level signifies the need to be courageous in life to make changes and pursue them without regrets.
	Level 3: Taking place on a dead planet, the difficulty is so high that LOL Inc. even used the Dark Artifact to prevent other players from clearing the level in order to monopolize the game world. But Wade Watts and his teammates, through wisdom and courage, finally managed to find the hidden cube and complete the level. This level not only tests players’ teamwork ability, but also signifies the importance of enjoying the process and going with the flow in life.
Reward mechanism	Rewards: For each level completed, players will receive a key to the final treasure, a recognition of their efforts and intelligence. And the ultimate reward is even more desirable - the power to gain control of the entire Oasis world, which represents supreme honor and freedom.
	Emotional Rewards: In addition to material rewards, players can also gain emotional satisfaction and growth during the clearance process. For example, Wade Watts learnt to be brave, persistent and cherished during the clearance process, and these qualities will stay with him for the rest of his life.

#### 3.3.2 Level tasks and rewarding mechanics in *Free Guy*

The level tasks and reward mechanism in *Free Guy* are the core of its narrative and thematic expression, which not only reflects the subversion of traditional game rules, but also deepens the film’s exploration of virtual and reality, free will and human nature, and the audience can feel a strong sense of immersion and achievement.

The specific content of the level tasks and reward mechanisms in *Free Guy* is shown in the table below (Table 2).

**Table 2:** List of level tasks and reward mechanics in *Free Guy.*

Level tasks/ reward mechanism	Content
Level tasks	Awakening and Self-Awareness: Guy gradually awakens from being an unconscious NPC and becomes aware of his own existence and the essence of the game world. This is the first ‘level’ he faces, requiring him to understand and accept his new identity and abilities.
	Fighting against the forces of evil: Guy encounters various evil forces and challenges in the game, including attacks from other players, unlawful behavior within the game, and so on. He needs to courageously stand up against these evil forces to protect himself and others.
	Finding Love and a Partner: Guy meets Millie in the game and falls in love with her at first sight. He needs to overcome a lot of obstacles to make a connection with Millie and face the challenges in the game together.
	Save the Game World: As the plot progresses, Guy and Millie discover the conspiracy behind the game world and decide to join forces to save it. This requires them to complete a series of arduous tasks, including uncovering the truth and confronting the boss of the game company.
Reward mechanism	Rewards for Regular Tasks: NPCs in the game have daily tasks that will be rewarded upon completion. For instance, if the main character Guy completes the bank teller job task every day, he will be rewarded with some basic rewards such as virtual currency, which is used to buy items such as coffee in the game world to maintain his daily life in the game.
	Reward for special missions: After Guy decides to become a ‘hero’, he starts to complete various special missions including rescuing others. For example, he saves people from robbers and completes missions that have special significance to the game world. Although the film does not clearly show the specific rewards for each mission, Guy gradually gains the respect and recognition of other characters in the game in the process of completing these missions, enhancing his status and influence in the game world. This sets the stage for his subsequent confrontation with the masterminds behind the game company, where he is able to get many NPCs to do their best to co-operate with him.
	Defeat Enemy Rewards: In the game’s combat scenarios, players are usually rewarded for defeating enemies with experience points, gold coins, equipment, and other rewards. For example, after fighting other ‘bad players’, Guy and his friends may be rewarded with in-game currency or better weapons and equipment, which will help them deal with challenges more easily in subsequent rounds of the game.
	Battle Achievement Rewards: If special achievements are reached in battle, such as winning with less, dodging enemy attacks perfectly, etc., one may be rewarded with special titles or props. The film does not directly reflect Guy getting these, but they are present in the game logic.
	Discovering New Locations Reward: Players exploring unknown areas of the game world may find hidden treasures, quest clues, or obtain special props. Guy discovers some eggs and hidden plots left by the game developers in the process of exploring the game world, which can be regarded as a kind of exploration rewards for him to understand the essence of the game world.
	Unlocking New Plot Rewards: As exploration progresses, players may unlock new plot lines and quest chains for a richer gameplay experience and more generous rewards. Guy unlocks a series of plots related to Milly, the main character, through continuous exploration, and eventually helps Milly find evidence to prove that the game company owner copied the idea, which in a sense is also a great reward from exploration.

#### 3.3.3 Level tasks and rewarding mechanics in *Everything Everywhere All at Once*

The task design and reward mechanism of *Everything Everywhere All at Once* reflects the exploration of the multiverse and the promotion of humane values, emphasising the significance of emotional satisfaction and self-realization. The design of these mission levels and reward mechanisms constantly stimulates the curiosity of the audience, engages them deeply emotionally and intellectually, and deepens the film’s exploration of family, self-identity and life choices.

The specifics of the level tasks and reward mechanisms in *Everything Everywhere All at Once* are shown in the table below (Table 3).

**Table 3:** List of level tasks and reward mechanics in *Everything Everywhere All at Once.*

Level tasks/ reward mechanism	Content
Level tasks	Laundry Universe: this is Evelyn’s main universe, where she manages a laundromat and faces tax issues and family conflicts. This mission is a solution to a real-life dilemma and symbolises her struggle between family and career.
	Chef Universe: In this universe, Evelyn is a chef tasked with solving problems through cooking. This task reflects her sense of responsibility to her family and her desire to take control of her life.
	Kung Fu Star Universe: In this universe, Evelyn is a Kung Fu star tasked with gaining power through fighting. This task symbolises her pursuit of self-worth and her desire for power.
	Hot Dog Fingers Universe: in this dystopian universe, Evelyn and her husband both have hot dog fingers and are tasked with solving problems through absurd behavior. This task reflects the film’s use of absurdity and humor, while also exploring the meaning of identity and existence.
	Stone Universe: In this universe, Evelyn and her daughter Joy are both stones tasked with understanding each other through silence and stillness. This task symbolises the emotional disconnect and communication barrier between her and her daughter.
Reward mechanism	Ability Rewards: Evelyn gains new abilities or skills after completing certain missions. For instance, in the Kung Fu Star Universe, she gained a strong fighting ability through battles, which helped her deal with challenges in other universes.
	Emotional Rewards: Evelyn gains emotional growth and understanding by completing certain quests. For instance, in the Stone Universe, she understands her daughter Joy’s inner world through silence and stillness, which helps her mend her relationship with her daughter.
	Knowledge Reward: Evelyn gains new knowledge about the multiverse and her own situation by completing certain quests. For example, she gradually understands how the multiverse works and her role and mission within it.
	Symbolic Rewards: Upon completion of certain tasks, Evelyn receives symbolic rewards that represent her new understanding of self and family. For example, her ultimate realization that her family is her most important universe is itself a symbolic reward.

## 4 A doctrinal analysis of the architecture of audiovisual immersiveness in gamified cinema

As a current cultural hotspot, ‘immersion’ has had a great impact on the field of art and communication. ‘Immersion’ has become the key word for creating art, broadening senses and creating experience, representing the new trend of popular aesthetics, and has become the aesthetic standard and tool for organising materials, bonding cultural psychology and structuring sensory experience, widely influencing the creation of contemporary films. Audiences are eager to establish a spiritual connection with the works that transcends time and space, and to obtain a richer aesthetic experience (Xu, 2022).

The audio-visual immersive design in gamified films is not only a technical tool, but also an important part of the film narrative. They blur the boundaries between film and game, creating a new narrative experience that retains the narrative depth of film while incorporating the interactivity and immersion of games, opening up new possibilities for film narrative.

### 4.1 Immersive design of visual scenes: spectralization effects and emotional evocation mechanisms

Gamified films focus on spectacle and immersion in visual presentation, integrating the virtual world with the real world through the setting of fantastical fight scenes, makeover scenes and special effects scenes, bringing the audience alternative satisfaction based on spectacle visual expression, with significant gamified characteristics. These scenes not only construct a whole different world with a unique visual style, but also transform into an immersive experience with both sensory impact and emotional projection, becoming an important part of the film’s narrative.

This study will analyze the fight scene of *Ready Player One*, the makeover scene of *Free Guy*’s and the special effects scene of *Everything Everywhere All at Once, respectively.*

#### 4.1.1 *Ready player one* fighting scene

**Figure 1:**
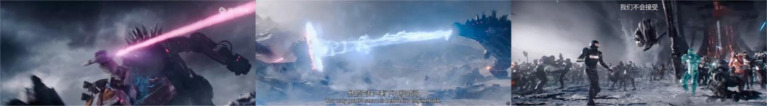
*Ready Player One* fighting scene.

Obviously, the fighting effects in the film are exaggerated and stunning, similar to the style of special effects designed to enhance the visual effects in the game. When the character attacks with a special weapon, it generates a dazzling light, explosion effects, and a lightsaber-like effect similar to what is shown in the first frame of Figure 1, reminiscent of sci-fi movie scenes. In the second frame, when the monster attacks, it emits blue flames and creates a large-scale energy wave explosion visual effect, which is both novel and full of a gaming vibe, as if the player is showcasing a cool special skill effect in the game.

Actually, the design of actions in fighting scenes has a certain sense of exaggeration and unreality, which is an important feature of camera language in gamified movies. For instance, characters’ movements sometimes exceed the physical limits of normal people, such as making long jumps and rolls in the air or moving and attacking at extremely fast speeds. The character’s movements sometimes exceed human physical limits, allowing for extended mid-air jumps, rolls, or incredibly fast movements and attacks, as shown in the third frame of Figure 1. This design aligns with the exaggeration of character actions in games to enhance fun and excitement, providing the audience with a game-like experience that transcends reality.

#### 4.1.2 *Free guy* makeover scene

**Figure 2:**
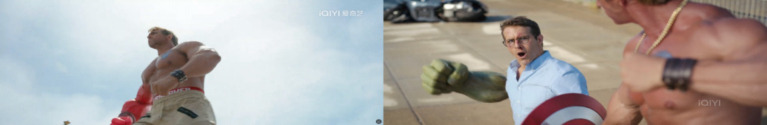
*Free Guy* makeover scene.

The makeover scenes in gamified films can successfully create an immersive experience, with stunning visual effects that create strong emotional resonance and psychological satisfaction for the audience. In *Free Guy*, the characters have the ability to change their equipments as the story takes place in a video game. During the confrontation between the main character, Guy, and the villain, Antoine, Antoine creates the character, Dude, who is able to transform in the middle of a battle, as shown in the first shot of Figure 2, where Dude transforms into a big man with strong muscles. Meanwhile, Guy, after using the Player Sunglasses, inspires the same transformation ability as Dude, and ultimately wins, as in the second shot of Figure 2 where Guy’s arm transforms into the mighty arm of the Hulk. These scenes not only show the power of the characters’ metamorphosis, but also make the audience feel as if they are personally involved in this fantastical adventure, making them some of the most engaging and immersive moments in the film.

#### 4.1.3 *Everything everywhere all at once* special effects scene

The function of special effects scenes in gamified films is irreplaceable. They are not only the core of visual appeal, but also an important means of narrative, emotional expression and immersion creation, which injects a unique charm into the film and makes it resonate deeply with the audience both visually and emotionally.

The special effects scenes in *Everything Everywhere All at Once* can be divided into two parts: those for the sets and environments, and the character-related special effects.

**Figure 3:**
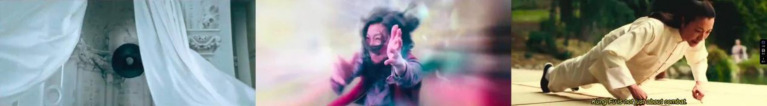
*Everything Everywhere All at Once* special effects scene.

In the sets and environments effects, the uniqueness of the film lies in the protagonist’s ability to jump back and forth between multiple parallel universes, resulting in rich and diverse scenes, including fantastical worlds like the Bagel World and the ancient world made of stones. For example, the Bagel World in Frame 1 of Figure 3 is a place created by Evelyn’s daughter, where the bagel carries everything in the world, eventually collapsing into a scene map resembling a black hole. The jumpability and transformation effects of such scenes align with the concept in games, where players can use special items to alter the environment or travel between teleportation points. This creates an experience for the audience, as if they are in a game world where they can freely interact and change things.

Among the character-related special effects, some possess special skills, and the design of special effects for these skills is also full of gameplay. For example, in character-related special effects, some characters possess unique skills, and the design of these skills’ effects is filled with a gaming vibe. For instance, after the protagonist Evelyn gains the ability to jump between universes, she could access various abilities from different versions of herself across these universes, such as martial arts, cooking, singing, etc., helping her overcome the challenges she faces. This allows the audience to more intuitively experience the character’s powerful abilities. In Figure 3’s Frame 2, Evelyn uses a tool that assists her in universe jumping, traveling through time and space. Countless light and shadow transformations appear on the screen, clearly showing the shifting scenes of time and space, enhancing the audience’s sense of immersion. Additionally, in Frame 3, when the protagonist faces danger, she jumps to the Kung Fu universe, gains martial arts abilities, and quickly defeats the villain with high combat strength. The combination of action and special effects makes the audience feel as if they are involved in the action across the multiverse, creating a game-like sense of immersion.

### 4.2 Auditory acoustic immersion design: based on spatial ambience creation mechanisms

In an era of speedy renewal, in which new visual images have given rise to countless fresh, aesthetic tendencies, cinema has had to search for new elements to attract audiences in a context full of popular audiovisual cultural contests. Therefore, in the 21st century (Jenkins, 2000), where aesthetic sensibilities are shaped by video games, cinema, as a fully integrated, mechanic, and sensory-mythological art of the new generation (Welsh, 1975), has become a trend to incorporate gamified audiovisual elements.

In the process of constructing a gamified narrative, in order to match the visual presentation, gamified films also adopt game-like sound effects in some auditory designs, which include ambient sound effects, background music and character sound effects, to enhance the audience’s sense of immersion and participation. These sound effects may change in accordance with the development of the film’s plot, complementing the visual images and working together to create a game-like atmosphere.

#### 4.2.1 Immersive design for *Ready Player One* audio effects

*Ready Player One* not only creates a stunning audio-visual feast through carefully choreographed environmental sound effects, contextually appropriate music, and vivid character sound effects, but also successfully brings the audience into the virtual game world of Oasis. Meanwhile, it also provides an excellent example for film sound design in creating an immersive experience.

In the world of ‘Oasis’, different scenes have very different ambient sound effects. In the cyberpunk city, the hum of electronic devices, the roar of flying cars, and the cacophony of people on the street corners are all intertwined. As Wade drives his race car through it, the sound of the wind from surrounding buildings, the roar of other vehicles’ engines, and the occasional collision come from all directions, making the audience feel as if they are in the busy and noisy streets of a futuristic city, greatly enhancing the sense of immersion. The film also makes extensive use of classic game music, which immediately evokes memories of the audience’s past gaming experiences and creates a strong emotional resonance when the music is played. The sound design of the characters in the film is delicate and precise. When Wade uses his lightsaber, the unique sound effect of the lightsaber turning on, the buzzing sound when he swings it, and the sound of its collision with objects are all clearly discernible, so that the audience can intuitively feel the power of the lightsaber and the state of its use, and immerse themselves in the intense atmosphere of the battle.

#### 4.2.2 Immersive design for *Free Guy* audio effects

*Free Guy* builds a diversified and realistic game world scene through environmental sound effects, background music, and character sound effects, accentuating the emotions and atmosphere, shaping characters with vivid vitality, and creating an all-round immersive design of sound.

In the battle scene, the design of ambient sound effects brings the tense and exciting atmosphere to the extreme. When an explosion occurs, the deafening sound of the blast is accompanied by a strong air wave sound effect, as if the audience can feel the power of the shockwave. In the fierce battle between Guy and his friend Buddy against the villain, the rich battle sound effects perfectly match with the intense images, immersing the audience in the tense and exciting battle atmosphere. The background music of Guy’s daily gaming life is dominated by relaxing and cheerful melodies. When he hums a little tune as he goes to work at the bank or greets passers-by on the street, the background music is fast-paced and energetic, using bright instrumental tones, such as piano and guitar, to play a pleasant melody. This style of music complements the relaxed, everyday atmosphere of the game world, allowing the audience to feel Guy’s simple and happy state of life while being immersed in the laid-back atmosphere of the game world. The sound design of the main character Guy’s character is extremely detailed, fully displaying his character traits and behavior. The sound of his daily walk, with its brisk and rhythmic pace, reflects his positive and optimistic character. When he discovers that the world he lives in is a virtual game world and decides to change his fate, his movement sound effects become more powerful, and every time he runs and jumps is accompanied by the sound of firm footsteps, demonstrating his inner bravery and determination.

#### 4.2.3 Immersive design for *Everything Everywhere All at Once* audio effects

*Everything Everywhere All at Once* enhances the realism and immersion of the virtual world through sound design, background music and voice prompts, creating an audio-visual experience that is both absurd and profound.

When Evelyn is engaged in a melee with multiple enemies, the combination of fast punching and kicking movements with powerful and realistic sound effects enhances the visual representation of the action, making the audience feel as if they were there in the tension and intensity of the battle. As Evelyn enters a bizarre universe with a body made up of hot dog fingers, the soundtrack’s style changes abruptly, with a brisk tempo and a bizarre melody full of absurdity. The jumping musical notes and unique combination of instruments fit perfectly with the dystopian scenes in this strange universe, immersing the audience in this over-the-top virtual world. Evelyn is given voice prompts as she travels through different universes to complete her tasks. In a technological universe, the voice prompts are cold, mechanical, and electronic, clearly informing Evelyn of the mission’s objectives, current progress, and the dangers she faces. The voice prompts are like playing an immersive game, where Evelyn receives commands from the system in the virtual world, and the audience is also immersed in it, as if they are also taking part in the adventures in the virtual universe, which enhances the sense of realism and immersion in the virtual world, and at the same time, strengthens the film’s absurd yet profound style.

## 5 Aesthetic transmutation through narrative strategies

As a product of the in-depth integration of film art and video games, the gamified cinema breaks the limitations of traditional film viewing with the help of rich and diversified narrative strategies, and progressively realises cross-media and immersive film viewing experiences. In this multi-dimensional and deep-level development process, gamified films have completed the change of aesthetic style from single to multiple, from traditional to immersive, and from entertainment dominant to artistic manifestation. The aesthetic transmutation brought about by this narrative strategy not only enriches the aesthetic connotation of the film but also reflects the change in the audience’s aesthetic demand and the innovative development of the film art itself, which brings infinite possibilities for the future of film art.

### 5.1 Aesthetic styles from monolithic to multifaceted

In the development process of gamified films, the innovation of narrative techniques is the crucial factor that drives its aesthetic style from single to multiple. The use of non-linear narrative, multi-clue narrative, circular narrative, meta-universe narrative, and other techniques has injected a brand new vitality and charm into gamified films. Non-linear narrative allows the story to switch back and forth between the past, present and future, making the film no longer a simple storytelling but a thinking challenge, giving gamified films an aesthetic style of depth and complexity. The multi-clue narrative unfolds multiple storylines under the same time and space, and these clues intertwine and influence each other, adding richness and interest to the gamified film and making the aesthetic style of the film more diverse. The circular narrative is based on the time cycle, and the characters keep experiencing the same events over and over again. This kind of narrative brings a tense and exciting sense of rhythm, and also contains thoughts about destiny and human nature, which makes the gamified film both entertaining and thought-provoking, and makes the style of the film more unique. Meta-universe narrative builds a huge and complex virtual universe or multiverse, each universe has a unique setting and story, this narrative technique brings visual spectacle and infinite imagination, making the aesthetic style of gamified films more sci-fi and fantasy. The use of these narrative techniques allows gamified films to bid farewell to a single aesthetic style and move toward diversified integration, bringing audiences a richer and more unique viewing experience and opening up a new direction for the development of film art.

### 5.2 Changing aesthetics from tradition to immersion

In recent years, with the advancement of virtual reality (VR), augmented reality (AR) and other technologies, gamified films have achieved revolutionary aesthetic changes through visual and auditory immersive design. In terms of image presentation, relying on advanced computer graphics technology, it builds a gorgeous and realistic dynamic world that complements the marvelous settings of the virtual game world, allowing the audience to intuitively feel the unique aesthetics of the intertwining of the virtual and the real. In terms of auditory presentation, the application of advanced technologies such as high-fidelity audio technology, 3D surround sound and Dolby Atmos creates a 360-degree immersive sound space, bringing the audience a smoother and more infectious auditory experience. From the simple production of special effects in the early days to the creation of immersive fantasy worlds today, gamified films, with their unique narrative appeal, have demonstrated how technological advances have revolutionized the aesthetics of cinema in all aspects.

### 5.3 From entertainment dominance to artistic manifestation

From entertainment dominance to artistic manifestation is an important dimension of the aesthetic transmutation brought about by the narrative strategy of gamified cinema. Early gamified cinema was mainly centred on entertainment elements that stimulated the senses, such as tense and exciting action scenes and fantastical and gorgeous visual effects, aiming to bring the audience an instant entertainment experience. Films at this period are rather like simple transplants of game elements, focusing on the compactness of the plot and the impact of the images to attract the audience’s attention. Nevertheless, with the development of the film industry and the improvement of the audience’s aesthetic level, gamified films have begun to seek for deeper artistic expression, no longer satisfied with superficial entertainment, but showing unique artistic charm through the in-depth excavation of the themes of human nature, society, science and technology, and so on. Creators try to combine game elements with profound themes, focusing on the construction of narrative structure, characterization, emotional expression and other artistic aspects, to create films with both game characteristics and artistic impact. While entertaining the audience, these works also convey profound ideological connotations and emotional resonance. In this way, the film wraps a profound emotional core under the entertaining fantasy shell, completing the sublimation from mere entertainment of the public to artistic expression, allowing the audience to comprehend the philosophy of life in the midst of laughter and emotion, and showing the depth of the narrative strategy for the aesthetic transformation of gamified films.

## 6 Conclusion

The narrative strategy of gamified films has had a profound impact on film creation and the film industry, which not only promotes the continuous innovation of film narrative structure and presentation methods, and promotes the expansion and integration of the film and game industries, but also changes the market pattern, attracts the attention of young viewers and gamers, and achieves excellent box office results.

With the blurring of the boundaries between games and films, the interaction between interactive film and narrative games will become more intense, and gamified films, as a product of the combination of the two, have great potential for development. Prospectively, the narrative strategy of gamified film will show a more diversified and innovative development. With the continuous breakthrough and wide application of cutting-edge technologies such as artificial intelligence, future gamified films may explore more complex and open narrative structures, further blurring the boundaries between films and games. In the meantime, the integration of gamified films with other art forms will also become a future trend. The in-depth integration with literature, theatre, music and other art forms will bring richer narrative resources and expression methods to gamified films. Therefore, gamified films also face a series of challenges, such as the balance between technology and narrative, the adaptation of audience acceptance and aesthetic habits, and the excavation of narrative depth and connotation.

This essay gives a more detailed overview of the narrative strategies of gamified films, but due to the length and perspective, it covers a limited number of films and does not explore some of the narrative dimensions in more depth. Future research not only needs to cover more gamified films of different styles and themes, and do deeper excavation of each narrative level, but also strengthen empirical research by comprehensively applying multidisciplinary theories of cinematography, game studies, psychology, and communication studies. Through audience feedback and market data, we can gain an in-depth understanding of the audience’s acceptance and actual demand for the narrative strategies of gamified films, so as to provide more solid theoretical support for the creation and development of gamified films.

## Data Availability

The original contributions presented in the study are included in the article/supplementary material, further inquiries can be directed to the corresponding author.
